# Supplementary figures and images for: Metabolomic study of marine Streptomyces sp.: Secondary metabolites and the production of potential anticancer compounds
Source: PLoS One. 2020 Dec 21;15(12):e0244385. doi: 10.1371/journal.pone.0244385 (PMC7751980; doi:10.1371/journal.pone.0244385)

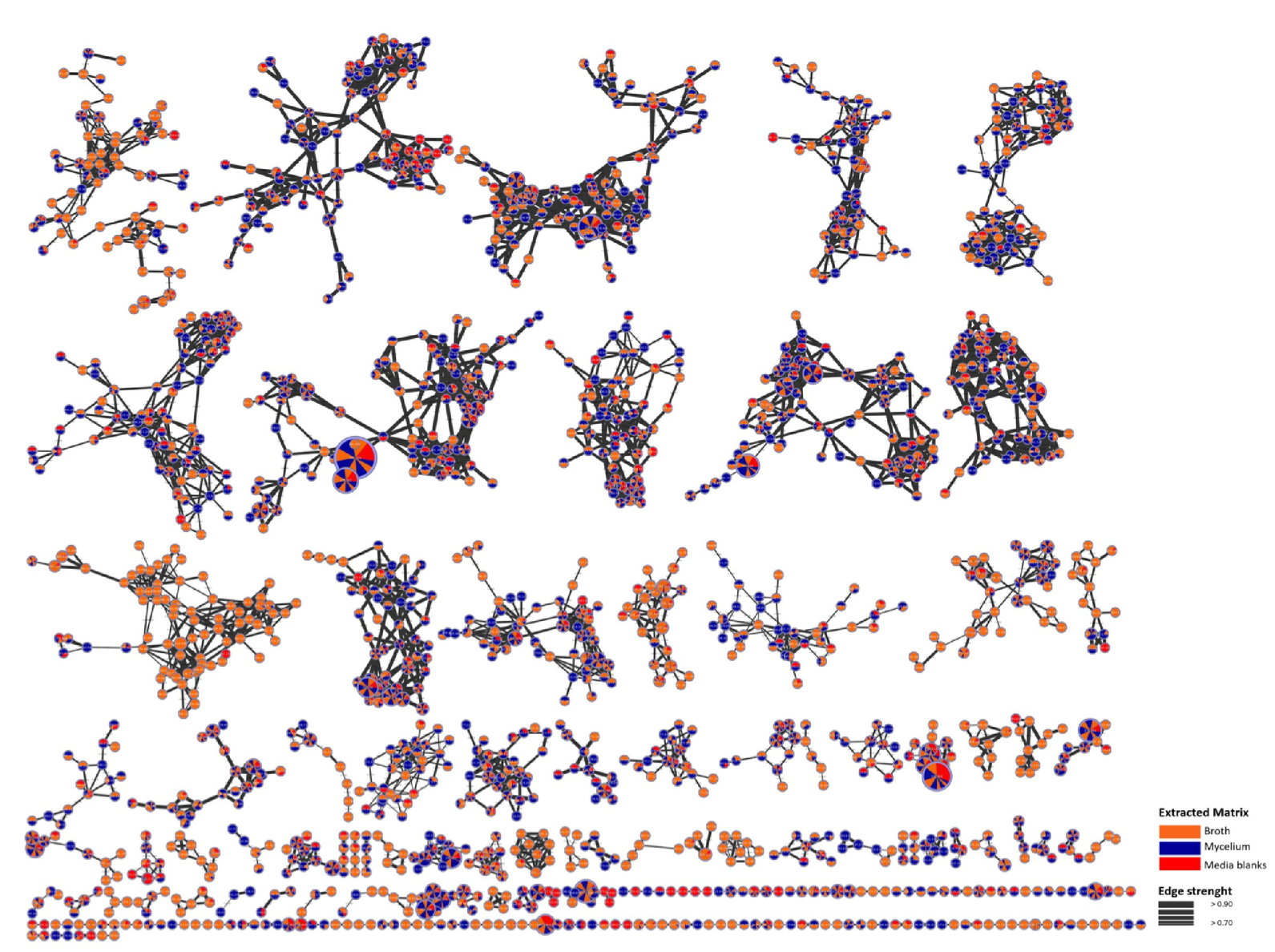

Supplement: S1 Fig — Six different media were used during seven days of cultivation. Nodes colored by extracted matrix. (TIF) [file pone.0244385.s001.tif]

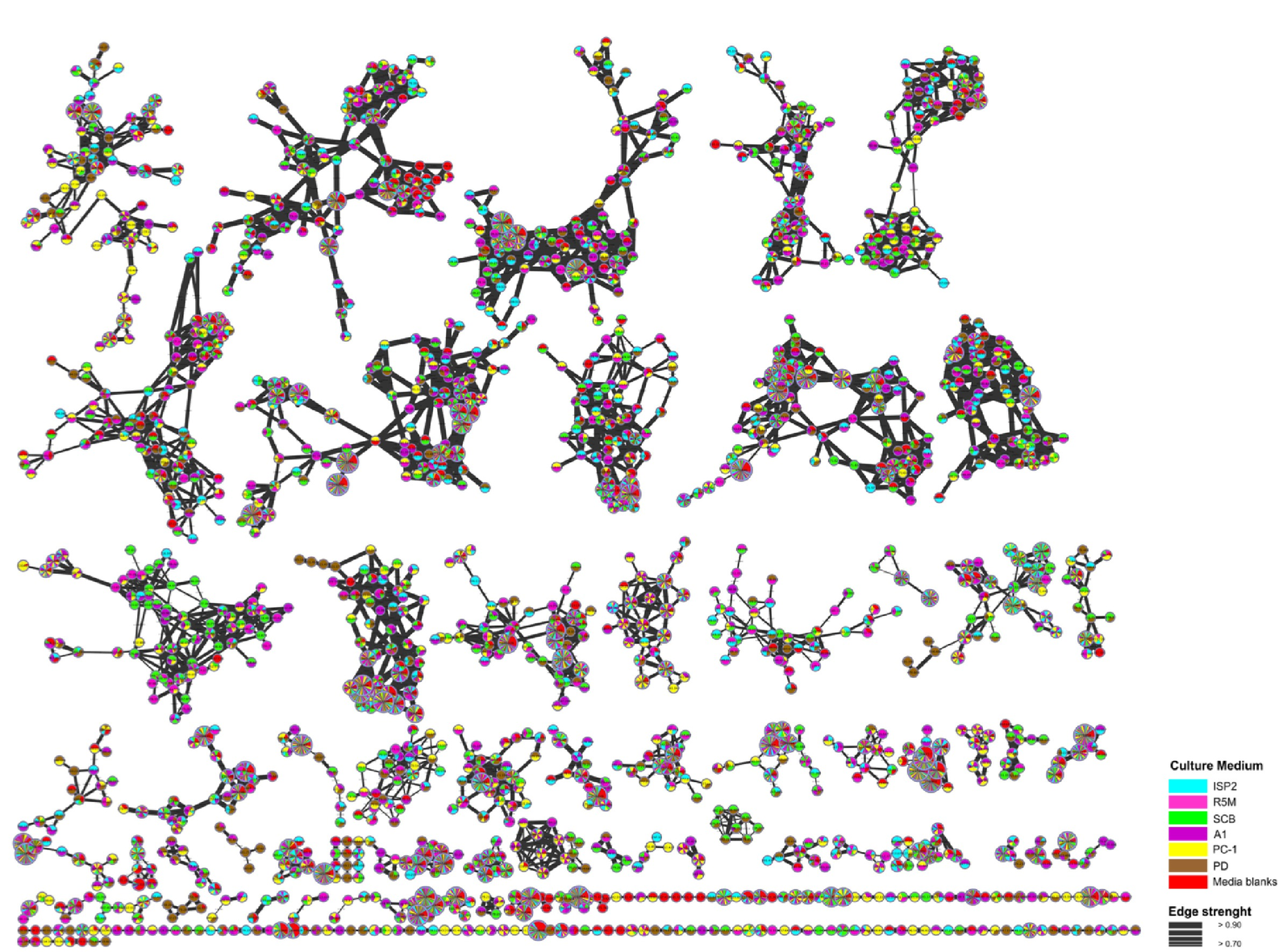

Supplement: S2 Fig — Six different media were used during seven days of cultivation. Nodes colored by extracted cultivation media. (TIF) [file pone.0244385.s002.tif]

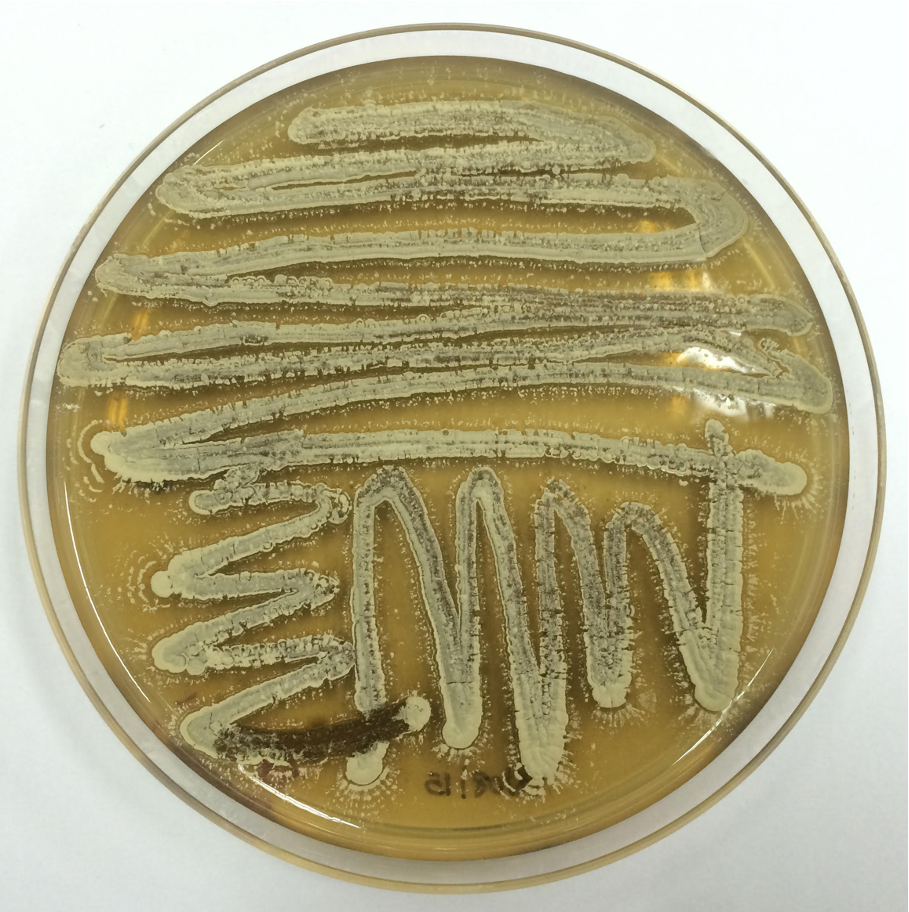

Supplement: S3 Fig — (TIF) [file pone.0244385.s003.tif]

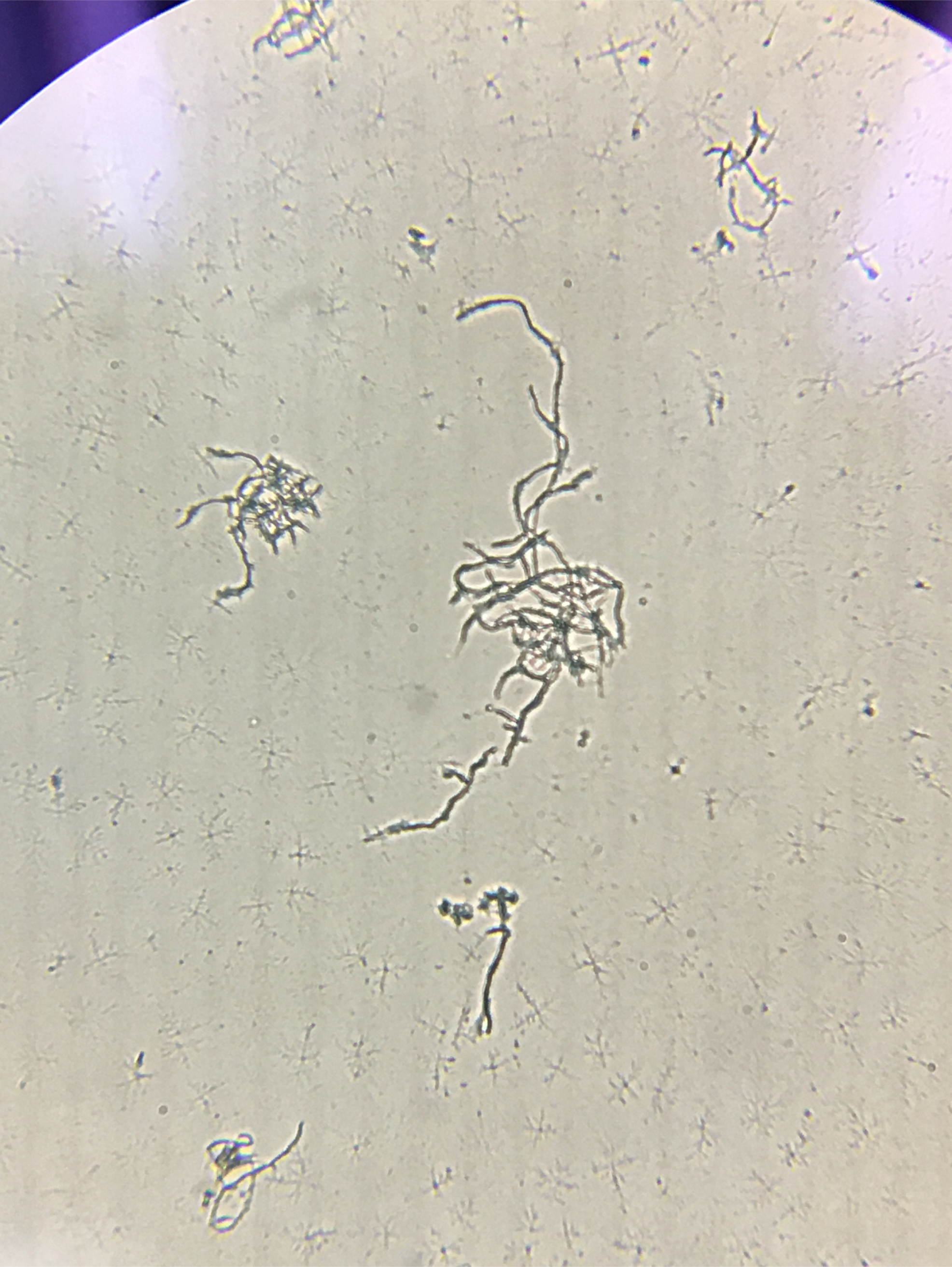

Supplement: S4 Fig — (TIF) [file pone.0244385.s004.tif]
