# Supplementary material for: Metabolomic study of marine Streptomyces sp.: Secondary metabolites and the production of potential anticancer compounds
Source: PLoS One. 2020 Dec 21;15(12):e0244385. doi: 10.1371/journal.pone.0244385 (PMC7751980; doi:10.1371/journal.pone.0244385)
Supplement: S1 Table — (DOCX) [file pone.0244385.s005.docx]

| ORF | Size  (aa) | Best BLASTp hit | ID (%) | Query  Cover (%) | BGC0001792 correlate | ID  (%) | Query  Cover (%) |
| --- | --- | --- | --- | --- | --- | --- | --- |
| 1 | 472 | Aldehyde dehydrogenase family protein  [*Streptomyces* sp. SID7982]  Access: NEE25902.1 | 99 | 100 | Aldehyde dehydrogenase | 91 | 100 |
| 2 | 269 | α/β-hydrolase  [*Streptomyces* sp. SID7982]  Access: NEE25903.1 | 99 | 100 | α/β-hydrolase | 90 | 100 |
| 3 | 84 | mbtH family protein  [*Streptomyces*]  Access: WP_100458032.1 | 100 | 100 | mbtH family protein | 93 | 100 |
| 4 | 265 | ABC transporter permease  [*Streptomyces*]  Access: WP_115068785.1 | 99 | 100 | ABC transporter permease | 96 | 100 |
| 5 | 339 | ABC transporter ATP-binding protein  [*Streptomyces*]  Access: WP_100458030.1 | 100 | 100 | ATP-binding cassette domain-containing protein | 87 | 92 |
| 6 | 123 | Hypothetical protein  [*Streptomyces*]  Access: WP_100458029.1 | 100 | 100 | Hypothetical protein | 91 | 100 |
| 7 | 138 | GntR regulator transcriptional  [*Streptomyces* sp. SID8014]  Access: WP_164378325.1 | 99 | 100 | GntR regulator transcriptional | 90 | 99 |
| 8 | 370 | Hypothetical protein  [*Streptomyces*]  Access: WP_100458027.1 | 99 | 100 | Membrane protein | 88 | 100 |
| 9 | 461 | Serine-hydrolase  [*Streptomyces griseus*]  Access: WP_115068786.1 | 99 | 99 | Serine hydrolase | 88 | 97 |
| 10 | 6449 | Nonribosomal peptide synthetase  [*Streptomyces griseus*]  Access: SUP57408.1 | 99 | 99 | Nonribosomal peptide synthetase | 68 | 89 |
| 11 | 1795* | Nonribosomal peptide synthetase [*Streptomyces diastaticus subsp. diastaticus*]  Access: GFH70652.1 | 99 | 100 | Nonribosomal peptide synthetase | 83 | 100 |
| 12 | 1633** | Nonribosomal peptide synthetase  [*Streptomyces griseus*]  Access: SUP57408.1 | 99 | 100 | Nonribosomal peptide synthetase | 86 | 100 |
| 13 | 6914 | Nonribosomal peptide synthetase [*Streptomyces diastaticus subsp. diastaticus*]  Access: GFH70652.1 | 91 | 92 | Nonribosomal peptide synthetase | 80 | 93 |
| 14 | 4114 | Hypothetical protein Sdia_14190  [*Streptomyces diastaticus subsp. diastaticus*]  Access: GFH70651.1 | 99 | 100 | Nonribosomal peptide synthetase | 82 | 100 |
| 15 | 529 | MFS transporter  [*Streptomyces*]  Access: WP_100455471.1 | 100 | 100 | MFS transporter | 92 | 100 |
| 16 | 430 | Hypothetical protein Srtu_15680  [*Streptomyces rutgersensis*]  Access: GFH65054.1 | 99 | 100 | Hypothetical protein | 61 | 93 |
| 17 | 329 | ABC transporter substrate-binding protein  [*Streptomyces* sp. SID8455]  Access: NEE43629.1 | 99 | 100 | ABC transporter substrate-binding protein | 90 | 100 |
| 18 | 254 | ABC transporter permease  [*Streptomyces*]  Access: WP_100455468.1 | 99 | 100 | ABC transporter permease | 88 | 88 |
| 19 | 238 | ABC transporter permease  [*Streptomyces*]  Access: WP_100455467.1 | 99 | 100 | ABC transporter permease | 95 | 95 |

* : 3´ truncated gene at right edge of contig X

** : 5´ truncated gene at left edge of contig Y

**S1 Table. Annotated ORFs for the predicted region as a cluster of surugamides biosynthetic genes in *Streptomyces* sp. BRB081.**
