# Supplementary material for: Metabolomic study of marine Streptomyces sp.: Secondary metabolites and the production of potential anticancer compounds
Source: PLoS One. 2020 Dec 21;15(12):e0244385. doi: 10.1371/journal.pone.0244385 (PMC7751980; doi:10.1371/journal.pone.0244385)
Supplement: Attachment — Submitted filename: Response to Reviewers.pdf [file pone.0244385.s006.pdf]

Universidade de São Paulo
Instituto de Biociências
www.ib.usp.br

São Paulo, 16th September 2020.

Dear Dr. Gupta,
Academic Editor
PLOS ONE

The manuscript is now revised and all suggestions provided by referees were implemented. These modifications are highlighted in the text with enabled "Track Changes" as required. Additionally, some specific questions from the reviewers were answered point by point below.

Additional requirements to address from the editor:

Question 1. Please ensure that your manuscript meets PLOS ONE's style requirements, including those for file naming. The PLOS ONE style templates can be found at https://journals.plos.org/plosone/s/file?id=wjVg/PLOSOne_formatting_sample_main_body.pdf and https://journals.plos.org/plosone/s/file?id=ba62/PLOSOne_formatting_sample_title_authors_affiliations.pdf

The manuscript was revised and instructions provided in both templates were strictly applied to meet PLOS ONE's style requirements.

Question 2. Please include captions for your Supporting Information files at the end of your manuscript, and update any in-text citations to match accordingly. Please see our Supporting Information guidelines for more information: http://journals.plos.org/plosone/s/supporting-information.

Captions for Supporting information were included at the end of the manuscript.

Question 3. We note that you have included the phrase "data not shown" in your manuscript. Unfortunately, this does not meet our data sharing requirements. PLOS does not permit references to inaccessible data. We require that authors provide all relevant data within the paper, Supporting Information files, or in an acceptable, public repository. Please add a citation to support this phrase or upload the data that corresponds with these findings to a stable repository (such as Figshare or Dryad) and provide and URLs, DOIs, or accession numbers that may be used to access these data. Or, if the data are not a core part of the research being presented in your study, we ask that you remove the phrase that refers to these data.

The phrase "data not shown" was removed since the data is not a core part of the research being presented.

Response to reviewer's questions:

Reviewer #1:
It is a good work which needs to be published in Plos One after suggested revisions. The manuscript revolves around the drugs derived from the marine bacteria.
Variation in the expression of surugamides with increase in time-points needs more discussion.
In addition to bacteria, please include a line of discussion on about the other marine endophytic microorganisms which serve as a great source of cytotoxic compounds. This information has been thoroughly discussed in following articles which may be cited.
1. Uzma et al., Endophytic Fungi-Alternative Sources of Cytotoxic Compounds: A Review, PMID: 29755344, DOI: 10.3389/fphar .2018.00309.

2. Pandey A. (2019) Pharmacological Potential of Marine Microbes. In: Arora D., Sharma C., Jaglan S., Lichtfouse E. (eds) Pharmaceuticals from Microbes. Environmental Chemistry for a Sustainable World, vol 28. Springer, Cham
3. Bramhachari P.V., Anju S., Sheela G.M., Komaraiah T.R., Venkataiah P., Prathyusha A.M.V.N. (2019) Secondary Metabolites from Marine Endophytic Fungi: Emphasis on Recent Advances in Natural Product Research. In: Singh B. (eds) Advances in Endophytic Fungal Research. Fungal Biology. Springer, Cham
Inclusion of Discussion section is essential to provide a comprehensive view to the readers about the interpretation of results and literature.
Typographical errors must be rectified

Additional discussion about the expression of surugamides was added in lines 353-364 of the final manuscript. Also, suggested references were included and discussion of other marine endophytes was improved in lines 477-490. Combined Results and discussion section was chosen following the guidelines of PLOS ONE. The authors believe that showing the discussion immediately after the results provides a better understanding of the work done.

Reviewer #2:
The work represented is quite extensive and elaborate; which has made my work as a reviewer quite easy. The data mining efforts along with biological activity verification's is the best way forward in the quest for unique compounds. Marine actinobacteria is one of most current area of research and hence kudoos for exploring it.
Having said that there are a few minor queries and suggestions from my part regarding the work.
(a) Why don't you add a supplementary file with respect to the biochemical profile and morphological characteristics of Streptomyces sp. BRB081 along with its morphological image. I advise you to do so given the uniqueness of this strain and because its not been identified to the species level

Pictures with the morphological characteristics of strain BRB081 were included in the Supporting Information (S3 and S4 Figs). Unfortunately, due to COVID-19 restrictions in our institution, the biochemical profile could not be done. However, the complete genome was deposited in the GenBank and can be found under the Accession Nº JACVQE000000000.

(b) The 16S rRNA gene sequence and whole genome analysis data availability in GenBank NCBI with its accession no is missing. Kindly add them for authenticity. Without this the data mining is irrelevant and future comparison studies and uniqueness will be less explored.

The information was included in the manuscript (*Streptomyces* sp. BRB 081) Accession Nº JACVQE000000000.

(c) Cyto-Toxicity studies has been carried out on colon adenocarcinoma cell line (HCT-116 ATCC CCL-247). Is there a reason only this particular cell lines were chosen. Kindly justify?

HCT-116 tumor cell line is one of the most used cell lines in screening programs along the world (Farnaes, La Clair & Fenical, *Org. Biomol. Chem.* 2014, **12**(3), 418-423; Trzoss et al., *PNAS* 2014, **111**(41), 14687-14692), and its use facilitate comparison with literature data. Besides, it has a moderate sensitivity to chemotherapeutic drugs, when compared to many solid tumor cell lines, generally allowing the selection of interesting extracts to pursue with chemical purification.

(d) Surugamides is the particular secondary metabolite highlighted in this study. Kindly compare this compound availability, quantification and activity among other streptomyces genera, especially amongst the marine actinobacteria globally.

Additional discussion comparing the expression of surugamides among other marine actinobacteria was added in lines 353-364 of the final manuscript.

(e)7th day old Streptomyces sp. BRB081 extracts has been explored extensively in this study. Is there a basis why only 7th day of culture inocula should be the one to be explored. What happens to the secondary metabolite secretions on an advanced days of incubation?

Cultivation for 7 days is a protocol of the lab and the interruption of that time can be done with fast-growing bacteria. The period of 7 days was selected because it has the highest ratio of colony yield (biomass) and extract yield without showing signs of culture decline and start of catabolism.

On behalf of all the authors, I am respectfully resubmitting the manuscript for your review. We sincerely hope that the changes we have made in response to the reviewer's comments satisfy your expectations for publication.

Your sincerely,

Dr. Marcelo M. P. Tangerina
marcelotangerina@usp.br
Institute of Biosciences
University of São Paulo – USP
São Paulo – SP, Brazil
