# Supplementary material for: Metabolomic study of marine Streptomyces sp.: Secondary metabolites and the production of potential anticancer compounds
Source: PLoS One. 2020 Dec 21;15(12):e0244385. doi: 10.1371/journal.pone.0244385 (PMC7751980; doi:10.1371/journal.pone.0244385)
Supplement: Attachment — Submitted filename: Response to Reviewers.pdf [file pone.0244385.s007.pdf]

USP Universidade de São Paulo
Instituto de Biociências
www.ib.usp.br

São Paulo, 24th November 2020.

Dear Dr. Gupta,
Academic Editor
PLOS ONE

The manuscript was once again revised and all the minor changes suggested were implemented. These modifications are highlighted in the text with enabled "Track Changes" as required. Reviewer #1 stated that all questions were adequately answered. The specific questions from Reviewer #2 were answered below.

Question 1. Here in abstract its written "extractions of mycelia and broth" (Line 24-25). But what kind of extraction is not specified. Is it solvent based secondary metabolite extraction? Then it should be mentioned. Also line 25 its written "crude extracts were analyzed"; what type of solvent based crude extract did u obtain? was it methanol extract or ethyl:acetone extract? Kindly specify, because such ambiguous statements are to be avoided.

The kind of extraction and the type of solvent based crude extract obtained were specified in the abstract as asked.

Question 2. Also one small concern the accession number provided in the manuscript "Streptomyces sp. BRB081 (GenBank accession number JACVQE000000000)" is not to be found in the NCBI GenBank database. Kindly re-verify this accession no. as this number provided might have been a temporary depository number.

This Whole Genome Shotgun project has been deposited at DDBJ/ENA/GenBank under the accession JACVQE000000000. The version described in this paper is version JACVQE010000000 and the number was replaced in the manuscript. The records will be available within a few days.

All modifications are highlighted in the text with enabled "Track Changes" as required.

On behalf of all the authors, I am respectfully resubmitting the manuscript for your review. We sincerely hope that the changes we have made in response to the reviewer's comments satisfy your expectations for publication.

I also would like to disclose that we set aside a budget for the publication fees from a project that ends on the 11th of December, 2020. If possible, it would be very important for us if we can have a final decision before this deadline. Due to the financial crisis triggered by COVID-19 in Brazil, we face several cuts in science and our budget is currently very limited and restrict.

Your sincerely,

Dr. Marcelo M. P. Tangerina
marcelotangerina@usp.br
Institute of Biosciences
University of São Paulo – USP
São Paulo – SP, Brazil
